# Corticosterone and pyridostigmine/DEET exposure attenuate peripheral cytokine expression: Supporting a dominant role for neuroinflammation in a mouse model of Gulf War Illness

**DOI:** 10.1016/j.neuro.2018.10.006

**Published:** 2018-10-16

**Authors:** Lindsay T. Michalovicz, Alicia R. Locker, Kimberly A. Kelly, Julie V. Miller, Zachary Barnes, Mary Ann Fletcher, Diane B. Miller, Nancy G. Klimas, Mariana Morris, Stephen M. Lasley, James P. O’Callaghan

**Affiliations:** a Health Effects Laboratory Division, Centers for Disease Control and Prevention, National Institute for Occupational Safety and Health, Morgantown, WV, USA; b Institute for Neuro-Immune Medicine, Nova Southeastern University, Ft. Lauderdale, FL, USA; c Miami Veterans Affairs Medical Center, Miami, FL, USA; d Department of Cancer Biology & Pharmacology, University of Illinois College of Medicine at Peoria, Peoria, IL, USA

**Keywords:** Gulf war illness, Corticosterone, Diisopropyl fluorophosphate, Pyridostigmine, DEET, Inflammation

## Abstract

Gulf War Illness (GWI) is a chronic multi-symptom disorder experienced by as many as a third of the veterans of the 1991 Gulf War; the constellation of “sickness behavior” symptoms observed in ill veterans is suggestive of a neuroimmune involvement. Various chemical exposures and conditions in theater have been implicated in the etiology of the illness. Previously, we found that GW-related organophosphates (OPs), such as the sarin surrogate, DFP, and chlorpyrifos, cause neuroinflammation. The combination of these exposures with exogenous corticosterone (CORT), mimicking high physiological stress, exacerbates the observed neuroinflammation. The potential relationship between the effects of OPs and CORT on the brain versus inflammation in the periphery has not been explored. Here, using our established GWI mouse model, we investigated the effects of CORT and DFP exposure, with or without a chronic application of pyridostigmine bromide (PB) and N,*N*-diethyl-meta-toluamide (DEET), on cytokines in the liver and serum. While CORT primed DFP-induced neuroinflammation, this effect was largely absent in the periphery. Moreover, the changes found in the peripheral tissues do not correlate with the previously reported neuroinflammation. These results not only support GWI as a neuroimmune disorder, but also highlight the separation between central and peripheral effects of these exposures.

## 1. Introduction

Nearly one-third of the veterans of the 1991 Persian Gulf War returned from duty with a chronic multi-symptom disorder, termed Gulf War Illness (GWI), experiencing fatigue, chronic pain, cognitive dysfunction, and headaches among many other symptoms (Fukuda et al., 1998; Steele, 2000; Smith et al., 2013; Dursa et al., 2016; White et al., 2016). The close association of these symptoms with those of “sickness behavior,” a physiological response to illness that has been associated with the elaboration of inflammatory cytokines in the brain (see Dantzer and Kelley, 2007), has supported a neuroinflammatory basis for GWI. Continuing research on the causes of GWI has pointed to the potential involvement of exposures to toxic chemicals during the war, including oil well fire smoke, pesticides, stress, nerve agent, and prophylactic drugs (Sullivan et al., 2003; Kerr, 2015; Research Advisory Committee (RAC) (2008); White et al., 2016; Sullivan et al., 2018). Previously, we have demonstrated that exposure to the organophosphate (OP), irreversible acetylcholinesterase (AChE) inhibitors diisopropyl fluorophosphate (DFP), as a sarin surrogate, and the pesticide chlorpyrifos results in neuroinflammation in rodents that is further exacerbated by prior chronic exposure to the stress hormone corticosterone at levels associated with high physiological stress (O’Callaghan, et al., 2015; Locker et al., 2017; Koo et al., 2018; Miller et al., 2018). While epidemiological studies have focused significant attention on the potential for the nerve agent prophylactic and reversible AChE inhibitor pyridostigmine bromide (PB) to contribute to GWI (Sullivan et al., 2003; Steele et al., 2012; Sullivan et al., 2018), our prior work found no neuroinflammatory effects of acute PB exposure (Locker et al., 2017; Miller et al., 2018) or chronic PB exposure combined with the insect repellant N,*N*-diethyl-meta-toluamide (DEET)(O’Callaghan et al., 2015).

While research into the underlying cause of GWI has largely indicated it to be a neuroimmune-based disorder (for example, see Craddock et al., 2015; Georgopoulous et al., 2017; O’Callaghan et al., 2016), exposure to nerve agents, pesticides, and other chemicals in theater would have been experienced through a variety of routes (e.g., inhalation, dermal, ophthalmic, etc.) that would have resulted in a systemic exposure to these chemicals. While systemic or peripheral exposures can certainly result in neuroinflammation, the lack of individual, specific exposure data for veterans suffering with GWI makes it difficult to determine not only what chemicals may have caused their illness, but also how any of these chemicals worked to instigate disease. In order to evaluate the potential systemic or peripheral inflammatory effects of GW-relevant toxicant exposures, we evaluated the expression of inflammatory cytokines in the liver and serum of mice we have previously reported to have a neuroinflammatory response to our model of GWI (O’Callaghan et al., 2015).

## 2. Materials and methods

### 2.1. Materials

The drugs and chemicals for this experiment were provided by the following sources: diisopropyl fluorophosphate (DFP), pyridostigmine bromide (PB), N,*N*-diethyl-meta-toluamide (DEET), and ethanol (Millipore Sigma, St. Louis, MO, USA);corticosterone [CORT (Steraloids, Inc., Newport, RI, USA)]. Materials used for additional tissue analyses were of analytical grade and purchased from various commercial sources.

### 2.2. Animals

Adult male C57BL/6 J mice (n = 4–5 mice per group), 8–12 weeks of age were purchased from Jackson Labs (Bar Harbor, ME). All procedures were performed within protocols approved by the Institutional Animal Care and Use Committee of the Centers for Disease Control and Prevention, National Institute for Occupational Safety and Health and the US Army Medical Research and Materiel Command Animal Care and Use Review Office, and the animal facility was certified by AAALAC International. Upon arrival, mice were individually housed in a temperature- (21 ± 1 °C) and humidity-controlled (50% ± 10) colony room that was maintained under filtered positive-pressure ventilation and a 12 h light (0600 EDT)/12 h dark cycle (1800 EDT). Mice were given *ad libitum* access to food (Harlan 7913 irradiated NIH-31 modified 6% rodent chow) and water.

### 2.3. Dosing and tissue preparation

The tissues analyzed in this study were collected from the same animals that provided brain tissues in the previously published neuroinflammation study (O’Callaghan et al., 2015). Briefly, mice received a single, subcutaneous injection of PB (2 mg/kg/day) and DEET (20 mg/kg/day) each day for 14 days. Starting on day 8, mice were given CORT (200 mg/L in 0.6% EtOH) in the drinking water for a total of 7 days. On day 15, mice received a single, intraperitoneal injection of DFP (4 mg/kg) and were killed by decapitation at 2, 6, 12 and 72 h post-DFP. Trunk blood was collected immediately upon decapitation for serum isolation using BD Microtainer Serum Separator tubes (BD365956; BD Biosciences, San Diego, CA, USA). Briefly, whole blood was allowed to clot for 10–15 minutes at room temperature and then centrifuged at 1000 × *g* for 15 min. The serum fraction was removed to a fresh tube and frozen at −80 °C until subsequent cytokine protein analysis. A portion of the liver was dissected free-hand and immediately frozen at −80 °C for subsequent RNA analysis.

### 2.4. Serum cytokine analysis

Twelve cytokines were measured in serum using Q-Plex™ Mouse Cytokine – Screen (16-plex), Quansys Imager, and Quansys reagents (Quansys Biosciences, Logan, Utah) at the E.M. Papper Laboratory of Clinical Immunology, Institute for Neuroimmune Medicine, Nova Southeastern University. Cytokine levels of IFNγ, IL-1α, IL-1β, IL-2, IL-4, IL-5, IL-6, IL-10, IL-12, KC, MIP-2, and TNFα were measured at 2, 6, 12, and 72 h post-DFP exposure and are expressed as pg/mL.

### 2.5. RNA isolation, cDNA synthesis, and qPCR

Total RNA was isolated from liver at 2, 6, 12 and 72 h after DFP exposure using methods previously described (Locker et al., 2017; Kelly et al., 2018). Real-time PCR analysis of the housekeeping gene, glyceraldehyde-3-phosphate dehydrogenase (GAPDH), and of the cytokines TNFα, IL-6, CCL2, IL-1β, leukemia inhibitory factor (LIF), oncostatin M (OSM), and IL-10 was performed in an ABI7500 Real-Time PCR System (Thermo Fisher Scientific, Waltham, MA, USA) in combination with TaqMan® chemistry as previously described (Locker et al., 2017; Kelly et al., 2018). Relative quantification of gene expression was performed using the comparative threshold (ΔΔC_T_) method. Changes in mRNA expression levels were calculated after normalization to GAPDH. The ratios obtained after normalization are expressed as fold change over corresponding saline-treated controls.

### 2.6. Statistical analyses

Prior to statistical analysis, data were analyzed for outliers using Grubb’s test with alpha set at 0.05 (GraphPad QuickCalcs: https://www.graphpad.com/quickcalcs/Grubbs1.cfm). Additional statistical analyses of the data were performed using SigmaPlot (v. 12.5; Systat Software, Inc). Within each time point, the test of significance was performed using two-way ANOVA (pretreatment [with PB/DEET v. without PB/DEET] × exposure [Saline, CORT, DFP, or CORT + DFP]) on log transformed values followed by multiple pairwise comparison analysis using Fisher least significant difference (LSD) post hoc test with statistical significance at 5% (p < 0.05). Data were log transformed as they did not follow a normal distribution. Graphical representations are of the mean ± SEM.

## 3. Results

**Fig. 1.**
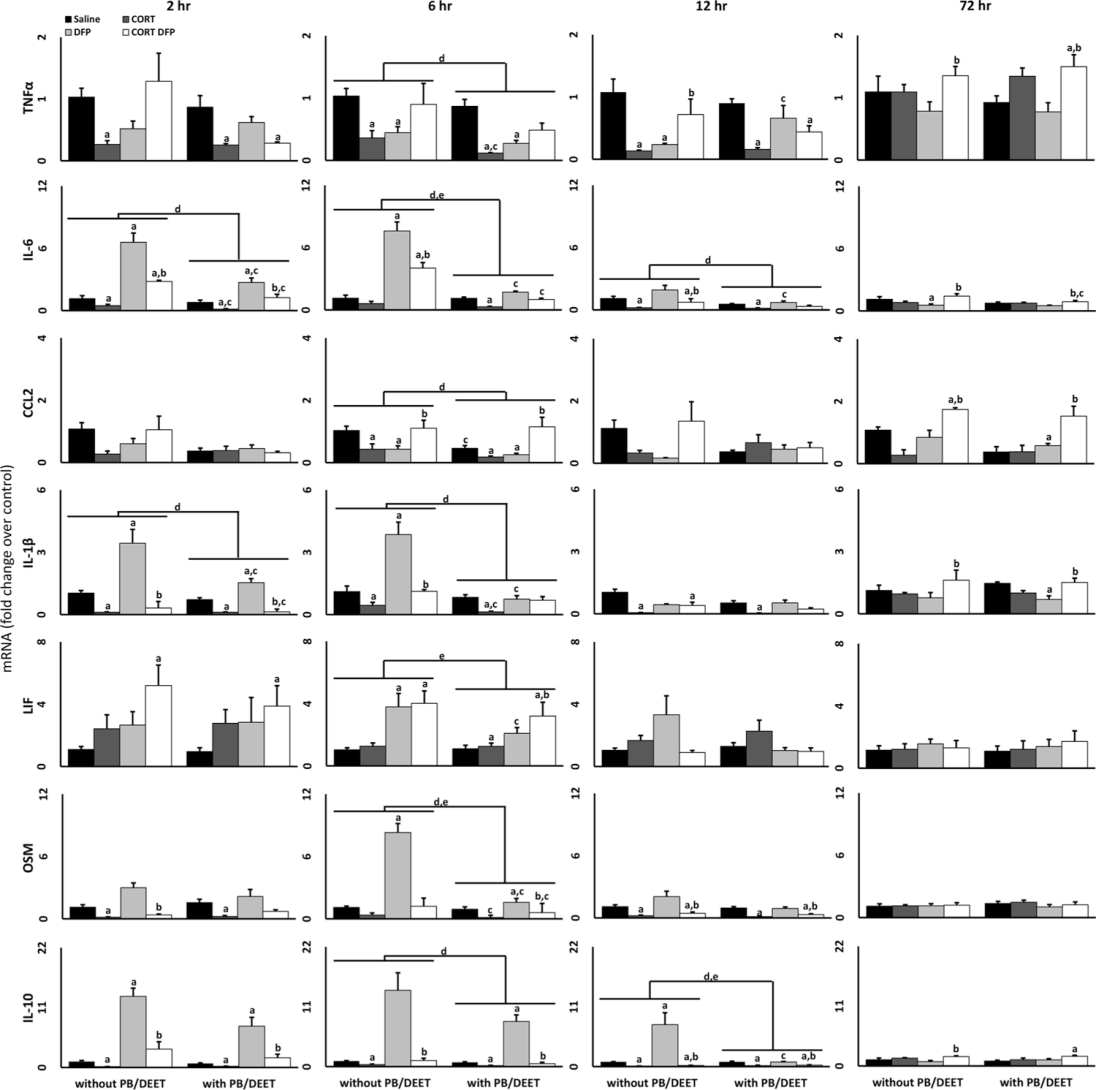
Cytokine expression in the liver of mice exposed to a GWI exposure model. Mice were exposed daily to PB (2 mg/kg/day, s.c.)/DEET (20 mg/kg/day, s.c.) for 14 days. Starting on day 8, the mice were administered CORT (200 mg/L) in the drinking water for 7 days followed by a single injection of DFP (4 mg/kg, i.p.) on day 15. TNFα, IL-6, CCL2, IL-1β, LIF, OSM, and IL-10 mRNA levels were measured in the liver of mice 2, 6, 12, and 72 h following DFP (or saline) exposure. Data represents mean fold change ± SEM (n = 4–5 mice/group). Statistical significance of at least p ≤ 0.05 is denoted by ^a^ as compared to saline (within pretreatment group), ^b^ DFP vs CORT + DFP (within pretreatment group), ^c^ as compared within exposure (without PB/DEET vs. with PB/DEET), ^d^ for a main effect of PB/DEET pretreatment, and ^e^ for a significant interaction between pretreatment and exposure.

**Fig. 2.**
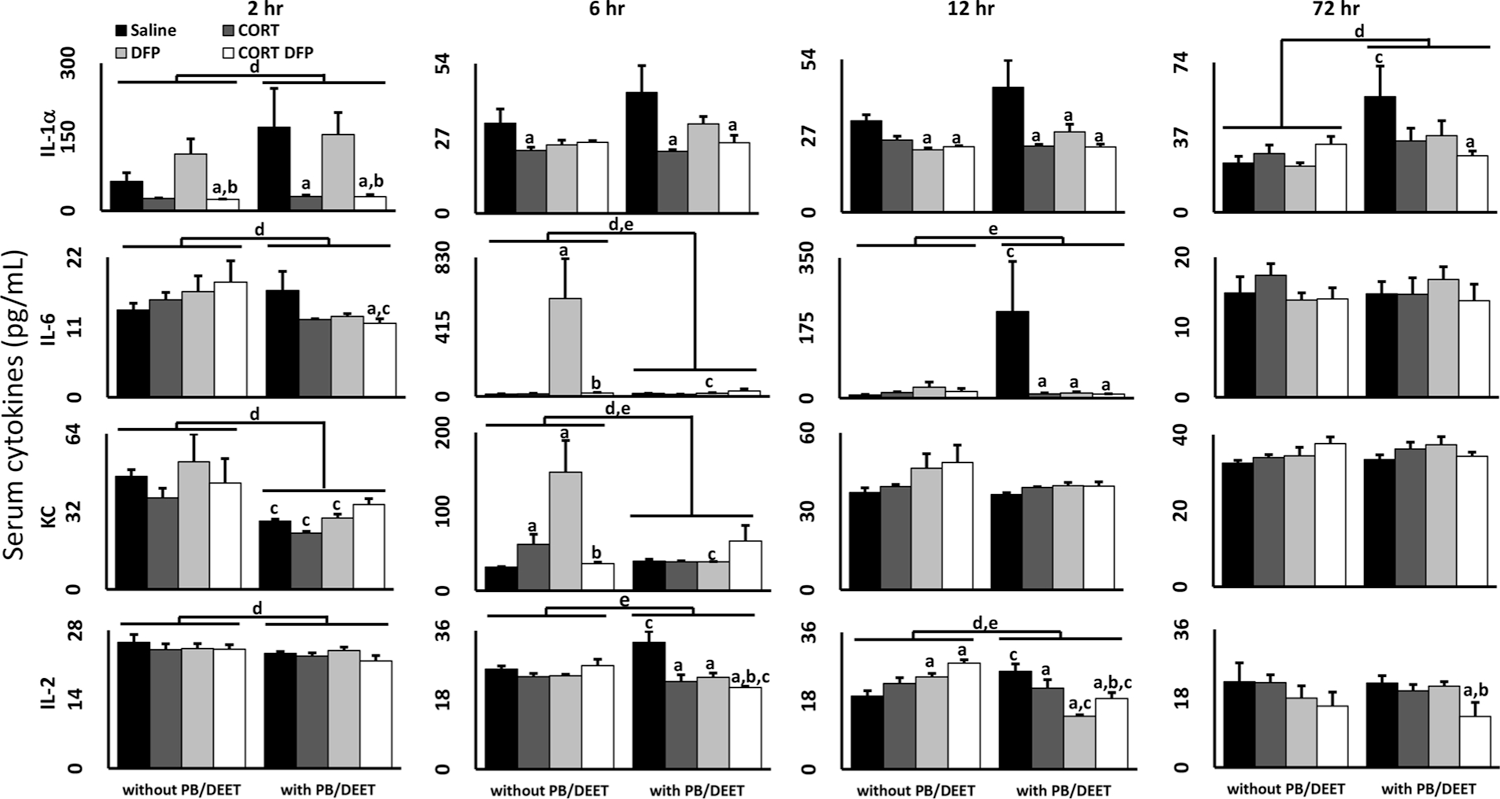
Proinflammatory serum cytokine levels in mice exposed to a GWI exposure model. Mice were exposed daily to PB (2 mg/kg/day, s.c.)/DEET (20 mg/kg/day, s.c.) for 14 days. Starting on day 8, the mice were administered CORT (200 mg/L) in the drinking water for 7 days followed by a single injection of DFP (4 mg/kg, i.p.) on day 15. Cytokine protein levels (pg/mL) were measured in the serum of mice 2, 6, 12, and 72 h following DFP (or saline) exposure. Data for IL-1α, IL-2, IL-6, and KC are shown and represents mean ± SEM (n = 4–5 mice/group). Statistical significance of at least p ≤ 0.05 is denoted by ^a^ as compared to saline (within pretreatment group), ^b^ DFP vs CORT + DFP (within pretreatment group), ^c^ as compared within exposure (without PB/DEET vs. with PB/DEET), ^d^ for a main effect of PB/DEET pretreatment, and ^e^ for a significant interaction between pretreatment and exposure.

**Fig. 3.**
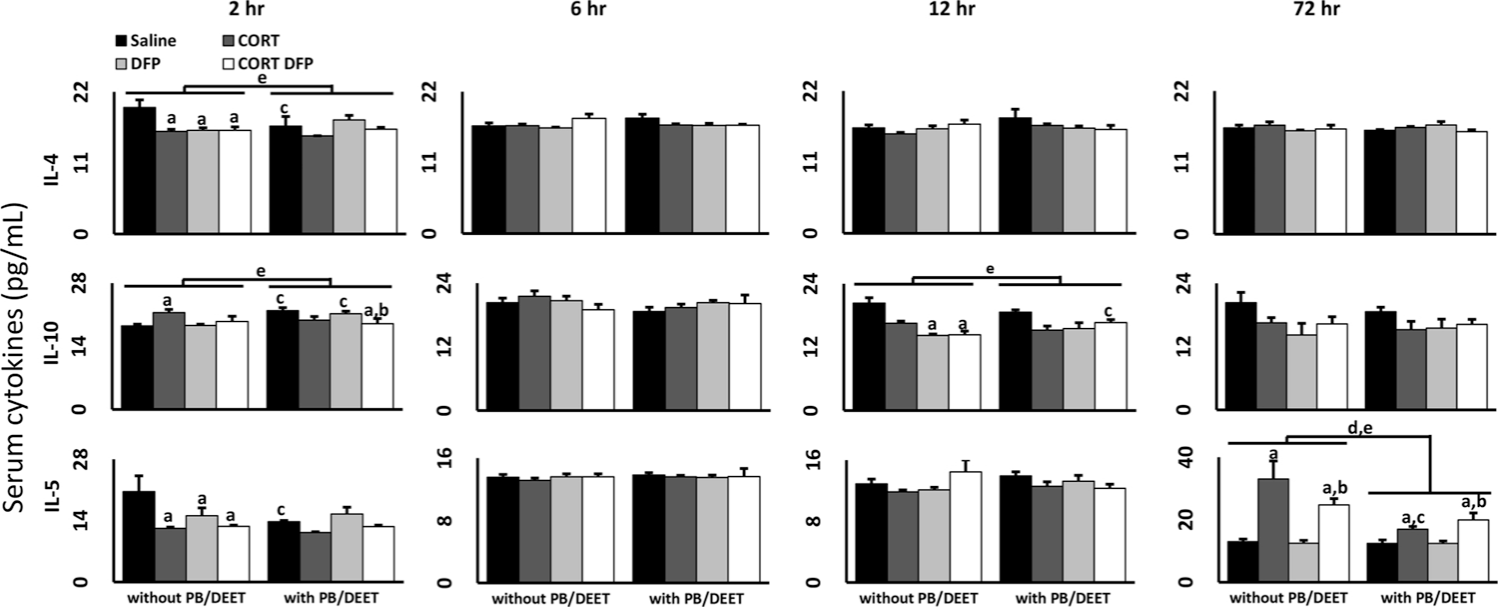
Anti-inflammatory serum cytokine levels in mice exposed to a GWI exposure model. Mice were exposed daily to PB (2 mg/kg/day, s.c.)/DEET (20 mg/kg/day, s.c.) for 14 days. Starting on day 8, the mice were administered CORT (200 mg/L) in the drinking water for 7 days followed by a single injection of DFP (4 mg/kg, i.p.) on day 15. Cytokine protein levels (pg/mL) were measured in the serum of mice 2, 6, 12, and 72 h following DFP (or saline) exposure. Data for IL-4, IL-5, and IL-10 are shown and represents mean ± SEM (n = 4–5 mice/group). Statistical significance of at least p ≤ 0.05 is denoted by ^a^ as compared to saline (within pretreatment group), ^b^ DFP vs CORT + DFP (within pretreatment group), ^c^ as compared within exposure (without PB/DEET vs. with PB/DEET), ^d^ for a main effect of PB/DEET pretreatment, and ^e^ for a significant interaction between pretreatment and exposure.

Previously, we have demonstrated that exposure to various GW-relevant toxicants can result in a marked neuroinflammatory response, particularly following combined exposure to chronic CORT and irreversible OP AChE inhibitors (O’Callaghan et al., 2015; Locker et al., 2017; Miller et al., 2018). Furthermore, we found that prolonged exposure to combined PB and DEET (O’Callaghan et al., 2015) or acute exposure to PB alone (Locker et al., 2017; Miller et al., 2018) had no proinflammatory effect in the brain. Considering that PB was used as a prophylactic against nerve agents to counteract the lethal peripheral symptoms of cholinergic toxicity (i.e. SLUDGEM) (Keeler et al., 1991; Research Advisory Committee (RAC) (2008)) and that many of the chemical exposures experienced during the war would have resulted in a whole-body exposure (i.e. inhalation, dermal exposure, etc), we wished to investigate the peripheral consequences of exposure in our GWI mouse model by examining inflammatory cytokines in the liver (Fig. 1) and serum (Figs. 2 and 3) of exposed mice. Evaluation of liver mRNA expression post-exposure found that DFP alone increased the expression of the pro-inflammatory cytokines IL-6, IL-1β, LIF, and OSM with little to no effect on TNFα and CCL2, but also greatly increased the expression of the anti-inflammatory cytokine, IL-10. These responses peaked at 6 h, similarly to what was previously observed in the brain tissue of these mice (O’Callaghan et al., 2015), with most cytokines returning to baseline levels by 12 or 72 h post-exposure. However, DFP significantly increased IL-6, IL-1β, and IL-10 expression as early as 2 h. Interestingly, this increased expression was significantly reduced by CORT exposure in all cases, contrary to our prior observations for CORT exacerbation of DFP-induced neuroinflammation (O’Callaghan et al., 2015; Locker et al., 2017), except for LIF which showed an exacerbated inflammatory response at 2 h. We also found a mild increase in TNFα and CCL2 cytokine mRNA expression at 72 h. Overall, PB/DEET exposure either had no effect or reduced the liver mRNA levels of the cytokines as compared to the groups that were not given PB/DEET pretreatment.

Of the twelve serum cytokines evaluated, only seven, including the anti-inflammatory cytokines IL-4, IL-5, and IL-10, demonstrated statistically significant changes over the time course evaluated (Figs. 2 and 3). For the proinflammatory cytokines (Fig. 2), DFP exposure alone resulted in marked increases in IL-6 and KC, an IL-8 homolog, at 6 h post-exposure and a minor increase in IL-2 at 12 h; for IL-6 and KC, CORT exposure ameliorated this inflammatory effect. Exposure to CORT with or without PB/DEET had an inhibitory effect, reducing the concentration of several cytokines relative to both saline-treated controls and the DFP alone group, except for IL-2. In general, PB/DEET exposure either had no effect or suppressed the response pattern observed without PB/DEET. However, PB/DEET exposure had a proin-flammatory effect on the concentrations of IL-1α at 2 and 72 h, IL-6 at 12 h, and IL-2 at 6 and 12 h.

The anti-inflammatory cytokines displayed various expression patterns over the time points evaluated (Fig. 3). At the earliest time point measured, both IL-4 and IL-5 were largely inhibited by all exposure conditions compared to saline alone. However, at this same time point, IL-10 was slightly elevated by CORT, PB/DEET alone, and PB/DEET + DFP, an effect that was lost at later time points. In particular, IL-10 expression was largely suppressed by all conditions at 12 h. While IL-4 demonstrated no significant changes beyond 2 h, IL-5 expression was largely increased by CORT exposure (CORT alone and CORT + DFP) with or without PB/DEET, though PB/DEET exposure dampened the response.

## 4. Discussion

Though the etiology of GWI is largely unknown, it has long been speculated that various toxic exposures experienced in theater may be the root cause of this sickness behavior-like illness (Sullivan et al., 2003; Steele et al., 2012; White et al., 2016; Sullivan et al., 2018). While many of the symptoms associated with GWI are largely neurological in nature, it is unclear whether these exposures have a direct effect on the central nervous system, or are the result of more systemic effects of these chemicals. Here, we have expanded upon our prior evaluation of the neuroinflammatory effects of exposure to GW-relevant toxicants (O’Callaghan et al., 2015; Locker et al., 2017; Koo et al., 2018; Miller et al., 2018) by investigating the potential peripheral effects of exposure to the OP sarin surrogate DFP, the nerve agent prophylactic PB, the insect repellant DEET, and the stress hormone CORT, to mimic high levels of physiological stress. In general, we found that DFP exposure alone was mildly proinflammatory in the serum, increasing the levels of proinflammatory cytokines IL-6 and KC (Fig. 2) and reducing the levels of the anti-inflammatory cytokines IL-4, IL-5, and IL-10 (Fig. 3), and liver, markedly increasing IL-6, IL-1β, LIF and OSM mRNA expression. However, DFP also resulted in a contradictory increase in the mRNA expression of the anti-inflammatory cytokine IL-10 in the liver. Contrary to our prior findings in the brains of these mice (O’Callaghan et al., 2015), chronic CORT exposure did not have a priming effect on DFP-induced inflammation in the liver (except for LIF at 2 h) or blood and even reduced the mRNA and protein expression of several cytokines. While CORT + DFP exposure did increase the mRNA expression of TNFα and CCL2 in the liver compared to DFP alone, these increases were relative to an inhibitory effect of DFP exposure on these cytokines and were not significantly different from control levels.

As addressed in previous publications (O’Callaghan, et al., 2015; Locker et al., 2017; Koo et al., 2018), the observation that CORT pre-treatment exacerbated DFP-induced neuroinflammation was surprising due to the generally anti-inflammatory effects of glucocorticoid signaling (Barnes, 2006; Coutinho and Chapman, 2011). However, in this study, we demonstrate that this phenomenon is largely exclusive to the brain as CORT exposure either had no effect on DFP-induced peripheral inflammation, or was inhibitory. Moreover, we found that CORT exposure alone increased the levels of the anti-inflammatory cytokines IL-5 and IL-10 in the serum, hinting at the anti-inflammatory nature of glucocorticoid exposure, at least in the periphery. These results also mirror our finding that exposure to chronic CORT has a protective effect on brain AChE activity, preventing the DFP-induced reduction in enzyme activity (Locker et al., 2017). This further suggests that while exposure to high physiological stress may have protected veterans with GWI from the immediate and potentially lethal central and peripheral toxicity of irreversible AChE inhibitors (e.g., sarin and chlorpyrifos), these same exposure conditions may have contributed to the chronic, neuroinflammation-based sickness behavior-like symptoms of GWI.

**Fig. 4.**
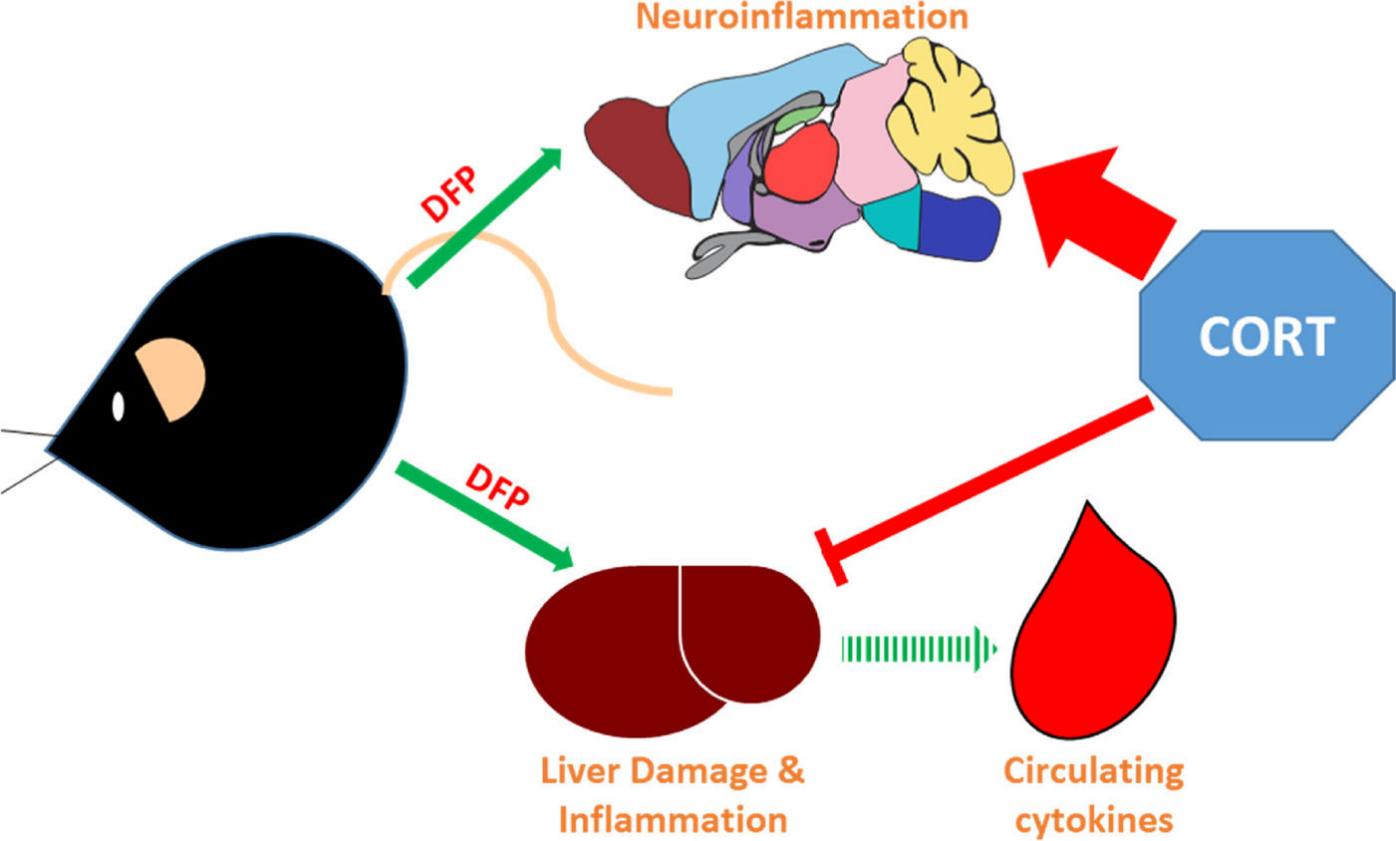
Effects of prior CORT exposure on brain and peripheral inflammation. Mice exposed to the organophosphate, AChE inhibitor DFP exhibit increases in proinflammatory cytokines in the brain and the liver. Unlike the brain, the inflammation observed in the periphery is coincident with liver damage and this inflammatory signaling increases the levels of circulating cytokines. Prior exposure to CORT primes the neuroinflammatory response to DFP, but inhibits the damage-induced inflammation in the liver and the secondary response in the blood.

While the neuroinflammatory priming effects of CORT exposure have been investigated previously (Frank et al., 2007, 2010; Munhoz et al., 2010; Loram et al., 2011; Frank et al., 2012), it is unclear why exogenous CORT has different effects on cytokine expression in the brain and peripheral tissues. One possibility is that CORT-induced inflammatory priming is unique to the brain and other tissues demonstrate a prototypical immunosuppressive response to the glucocorticoid. However, using the prototypical inflammagen lipopolysaccharide, chronic exogenous CORT exposure primes the LPS-induced neuroinflammatory response (Kelly et al., 2018), as well as the response in serum (O’Callaghan, unpublished data). This observation suggests that the more brain-exclusive inflammatory priming we find in our GWI model may be specific to DFP, and other OP exposures. However, OP compounds have been identified to cause liver damage and associated inflammation (Mostafalou et al., 2012; Lasram et al., 2014; Ezzi et al., 2016), supported by the observed increases in IL-6, IL-1b, LIF, and OSM mRNA in the liver and IL-6 and KC levels in the serum, though these responses were generally suppressed by CORT. Our hypothesis is that exposure to DFP directly instigates neuroinflammation that can be primed by CORT, as we have observed no indicators of neuronal damage in these mice (O’Callaghan et al., 2015), while the peripheral cytokine responses are the result of liver damage-induced inflammation that is non-responsive to prior CORT exposure (Fig. 4). In support of this hypothesis, we have found that chronic CORT has an inhibitory effect on the damage-induced neuroinflammation associated with exposure to the dopaminergic neurotoxicant, 1-methyl-4-phenyl-1,2,3,6-tetrahydropyridine (MPTP) (Michalovicz et al., 2015). However, CORT does prime these responses following exposure to methamphetamine (Kelly et al., 2012). Though these observations were made in brain tissue, they highlight the potential for different damage-induced inflammatory reactions to be differentially effected by exogenous CORT exposure. Thus, we suggest that the increased inflammation seen in the liver is the result of DFP-induced tissue damage that is suppressible by CORT exposure; the inflammatory response we observe in the serum of these animals is secondary to the liver damage/inflammation signaling due to its limited scope and delayed response (Fig. 4). For example, IL-6 mRNA is increased in the liver as early as 2 h post-DFP, but this same cytokine is not increased in the serum until 6 h after exposure.

Similarly to CORT exposure, PB/DEET pretreatment also generally had no or a mildly inhibitory effect on the cytokine profile in the liver and serum, results consistent with those observed for brain tissue from the same mice (O’Callaghan et al., 2015). While epidemiological studies have indicated a strong correlation between PB consumption and GWI (Sullivan et al., 2003; Steele et al., 2012; Sullivan et al., 2018), a similar assertion is difficult to make with our mouse model of GWI. As AChE inhibitors should activate the cholinergic anti-inflammatory pathway (Pavlov et al., 2003; Pavlov and Tracey, 2005; Pohanka, 2014), and PB has been shown to have anti-inflammatory effects in other illness models, such as those involving myocardial infarction and hypertension (Machado et al., 2012; Feriani et al., 2017; Bezerra et al., 2017; Feriani et al., 2018; da Silva et al., 2018), it is not surprising that PB would have a suppressive effect on cytokine expression. However, PB/DEET exposure alone did increase the expression of a few proinflammatory cytokines in the serum (IL-1α, IL-2, and IL-6; Fig. 2) and reduced or tempered the expression of anti-inflammatory cytokines in the liver (IL-10; Fig. 1) and serum (Fig. 3) at specific time points. While these conditions would support a more proinflammatory effect of PB/DEET, these responses occurred across different time points post-exposure unlike the coordinated, dramatic increase in cytokine mRNA expression observed in the brains of the same mice (O’Callaghan et al., 2015), making the influence of these responses on a more general inflammatory state unclear. Furthermore, outside of Gulf War Illness research, PB has not been positively associated with inflammation, though one myasthenia gravis case study reported GWI-like joint and muscle pain with PB treatment (Rostedt and Stålberg, 2004); while DEET has the potential to cause some inflammatory responses, this seems to be related to very high doses of exposure (DEET, 2016).

Many of the studies investigating the consequences of Gulf War Illness-relevant exposures in animal models combine PB and DEET with pesticide exposures, but have not evaluated the individual contribution of PB, DEET, or pesticide to their observed responses (Parihar et al., 2013; Zakirova et al., 2017; Shetty et al., 2017; Kodali et al., 2018; Petrescu et al., 2018; Seth et al., 2018). Thus, it is impossible to separate the responses to the individual agents as we have shown for the liver, serum, and brain (Figs. 1–3; O’Callaghan et al., 2015). While the use of PB has been shown to have a strong, general association with GWI in the afflicted veteran population (Steele et al., 2012), other studies have specifically found associations between PB usage alone or in combination with pesticide exposure and cognitive dysfunction (Sullivan et al., 2003, 2018). Based on our findings, it is possible that this association may be specific to cognitive impairment and not neuroinflammation or may not be apparent acutely following exposure to PB, considering that veterans with GWI are evaluated decades following their time in theater.

While these observations paint a complicated picture of the consequences of these exposures, evaluation specifically of the effects of combined exposure to PB/DEET and CORT shows this condition to result in greatly exacerbated DFP-induced neuroinflammation (O’Callaghan et al., 2015) with minimal and potentially inhibitory effects on the expression of cytokines in peripheral tissues. Overall, our results further support the notion that GWI is largely a neuroimmune illness instigated by exposure to several conditions experienced during the 1991 Persian Gulf War, particularly high physiological stress and irreversible AChE inhibitors.
